# WRKY6 restricts *Piriformospora indica*-stimulated and phosphate-induced root development in Arabidopsis

**DOI:** 10.1186/s12870-015-0673-4

**Published:** 2015-12-30

**Authors:** Madhunita Bakshi, Khabat Vahabi, Samik Bhattacharya, Irena Sherameti, Ajit Varma, Kai-Wun Yeh, Ian Baldwin, Atul Kumar Johri, Ralf Oelmüller

**Affiliations:** Institute of General Botany and Plant Physiology, Friedrich-Schiller-University Jena, Dornburgerstr. 159, D-07743 Jena, Germany; Max-Planck-Institute for Chemical Ecology, Beutenberg Campus, Hans-Knöll-Straße 8, D-07745 Jena, Germany; Amity Institute of Microbial Technology, AUUP, Noida, India; Institute of Plant Biology, Taiwan National University, Taipei, Taiwan; School of Life Sciences, Jawaharlal Nehru University, New Delhi, 110067 India

**Keywords:** WRKY6, *Piriformospora indica*, Phosphate, Root development, Expression profiles

## Abstract

**Background:**

Arabidopsis root growth is stimulated by *Piriformospora indica*, phosphate limitation and inactivation of the WRKY6 transcription factor. Combinations of these factors induce unexpected alterations in root and shoot growth, root architecture and root gene expression profiles.

**Results:**

The results demonstrate that *P. indica* promotes phosphate uptake and root development under Pi limitation in *wrky6* mutant. This is associated with the stimulation of *PHOSPHATE1* expression and ethylene production. Expression profiles from the roots of *wrky6* seedlings identified genes involved in hormone metabolism, transport, meristem, cell and plastid proliferation, and growth regulation. 25 miRNAs were also up-regulated in these roots. We generated and discuss here a list of common genes which are regulated in growing roots and which are common to all three growth stimuli investigated in this study.

**Conclusion:**

Since root development of *wrky6* plants exposed to *P. indica* under phosphate limitation is strongly promoted, we propose that common genes which respond to all three growth stimuli are central for the control of root growth and architecture. They can be tested for optimizing root growth in model and agricultural plants.

**Electronic supplementary material:**

The online version of this article (doi:10.1186/s12870-015-0673-4) contains supplementary material, which is available to authorized users.

## Background

Phosphorus (P) is an essential macronutrient for plant growth and development, making up to 0.2 % of the plant’s dry mass. P is involved in the regulation of many key metabolic pathways in all living organisms, including energy generation, nucleic acid and membrane synthesis, protein phosphorylation and redox reactions [1–3]. Plants absorb P from soil in the form of inorganic phosphate (Pi). Due to low availability and poor mobility [4, 5], the concentration of Pi in soil solutions is usually ~10 μM, which is below the critical level needed for the optimal performance of crops and plants [6]. It is estimated that ~5.7 billion hectares of land are deficient in P which can be mitigated by the application of fertilizers. Pi fertilization can cause ecological problems such as eutrophication or toxic algal blooms [7]. Soluble Pi in the soil also forms complexes with cations like calcium, magnesium, aluminum or iron, which are not readily absorbed by plants. Although plant growth-promoting rhizobacteria and fungi [e.g. arbuscular mycorrhizal (AM) fungi] enhance Pi uptake into the roots, microbes and weed also compete with plants for Pi, or convert it into organic forms that are not available to support plant growth [8, 9].

To cope with Pi limitations, plants have evolved complex adaptive responses that include morphological and physiological modifications to improve Pi acquisition or remobilization *via* the differential expression of various Pi transporter genes [10, 11]. Remodeling of root architecture, inhibition of primary root length, increase of root hair density and length, as well as associations with AM or AM-like fungi are typical developmental responses to low Pi [2, 12]. The role of the AM symbiosis in enhancing P acquisition from soils is well known [13].

*Piriformospora indica*, a mycorrhiza-like fungus, enhances growth of monocots and dicots [14–16]. The fungus improves nutrition uptake from the soil to the host roots [17, 18] which also includes Pi transfer *via* fungal hyphae through the high-affinity Pi transporter PiPT localized to the external hyphae [19]. PiPT is highly homologous to the *Saccharomyces cerevisiae* high-affinity Pi transporter Pho84 and to plant Pi transporters (cf. [20]). In addition to stimulating Pi metabolism, *P. indica* also enhances the expression of genes for nitrate reductase and the starch-degrading enzyme glucan-water dikinase in Arabidopsis roots [17] suggesting a strong fungal influence on the plant primary metabolism. To what extend this is responsible for the benefits of the plants in their symbiotic interaction with *P. indica* is not clear. In addition to increasing the plant’s biomass [21–23], enhanced resistance to biotic and abiotic stress [24, 25], the induction of systemic and local resistance [26, 27] and the stimulation of secondary metabolite accumulation [28] have been reported. This requires a highly balanced symbiosis in which the plants appear to control the degree of root colonization [29, 30].

WRKYs are important transcription factors (TFs) of the plant signaling web which regulate many responses to biotic and abiotic stimuli, but these TFs are also involved in responses to internal signals which coordinate developmental processes. They interact with DNA- and non DNA-binding proteins [31] and function as activators and repressors of gene expression, depending on their interaction partners and target genes [32, 33]. WRKY6, WRKY42 and WRKY75 are induced during Pi deprivation [34–36]. Chen et al. [34] showed that WRKY6 is involved in the response to low-Pi stress by regulating *PHOSPHATE1* (*PHO1*) expression. Low Pi treatment reduced WRKY6 binding to the *PHO1* promoter, which indicates that *PHO1* regulation by WRKY6 is Pi-dependent and that low Pi levels prevent inhibition of *PHO1* expression. The plant-specific WRKY75 is an activator of several Pi starvation-induced genes encoding phosphatases, Mt4/TPS1‑like proteins or high affinity Pi transporters [36]. Suppression of *WRKY75* expression through RNAi silencing induces stress responses, such as anthocyanin accumulation [35].

We noticed that *wrky6* seedlings and plants exposed to Pi limitation perform much better in the presence of *P. indica* when compared to the WT controls. *P. indica* also stimulated Pi uptake and translocation into the plant under Pi limitation, and these processes are restricted by WRKY6. The strong promotion of root development of *wrky6* plants exposed to *P. indica* under Pi limitation motivated us to perform comparative expression profiles to identify genes, proteins, as well as metabolic and signaling pathways which optimize root development, especially under Pi limitation conditions.

## Results

### Root phenotype of WT and *wrky6* seedlings under different Pi concentrations

**Fig. 1:**
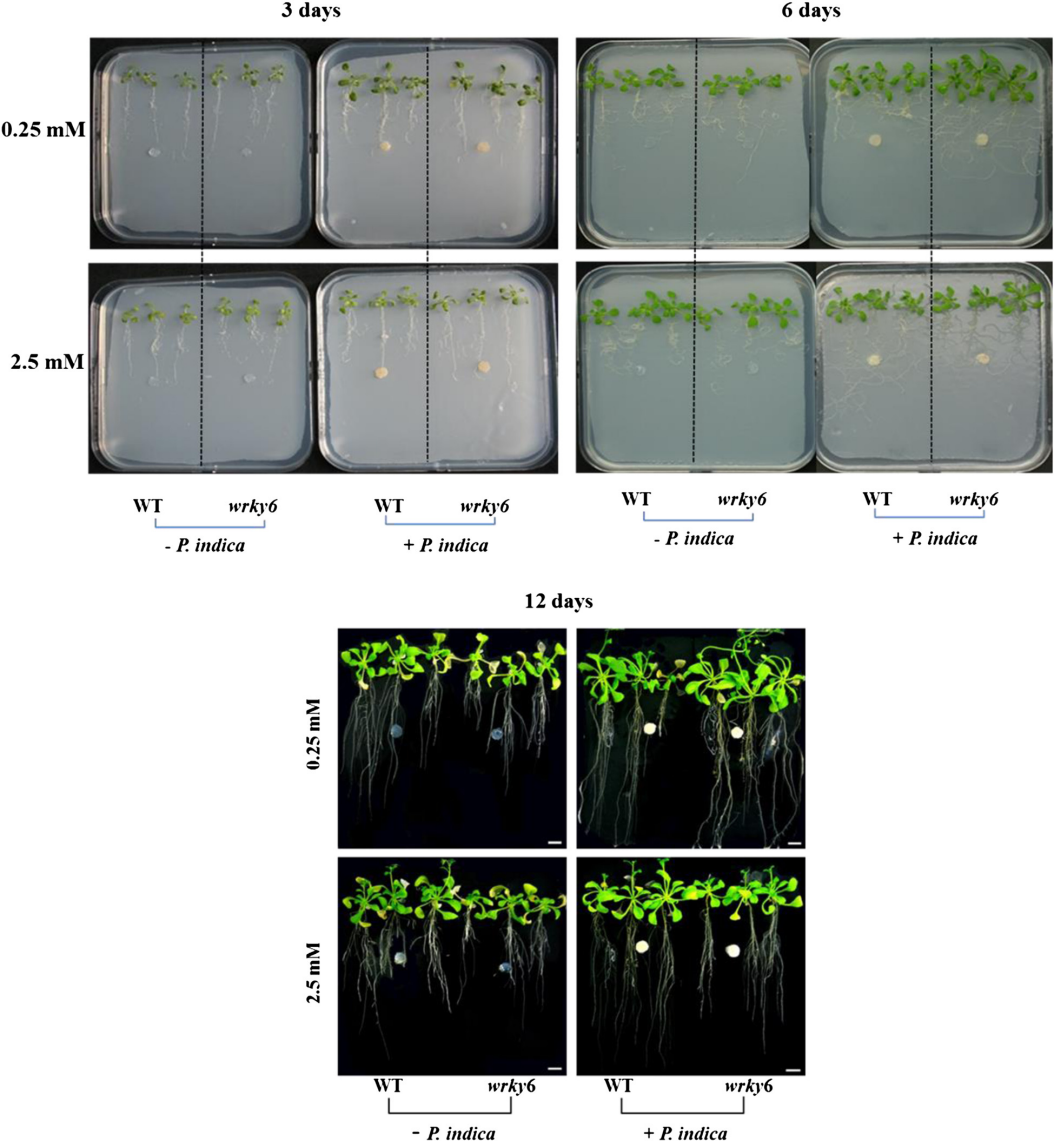
Phenotypes of WT and *wrky6* seedlings grown on 2.5 mM and 0.25 mM Pi in the medium. 10 day-old seedlings grown on MS medium were transferred to PNM media containing the two different Pi concentrations for additional 3, 6 and 12 days, either in the presence of *P. indica* (*right panels*) or plaques without the fungus (*left panels*). All seedlings were grown at 22 °C and 70–80 % humidity in a 16-h light/8-h dark cycle. Photos show typical view of more than 10 repetitions. Bar: 1 cm

**Fig. 2:**
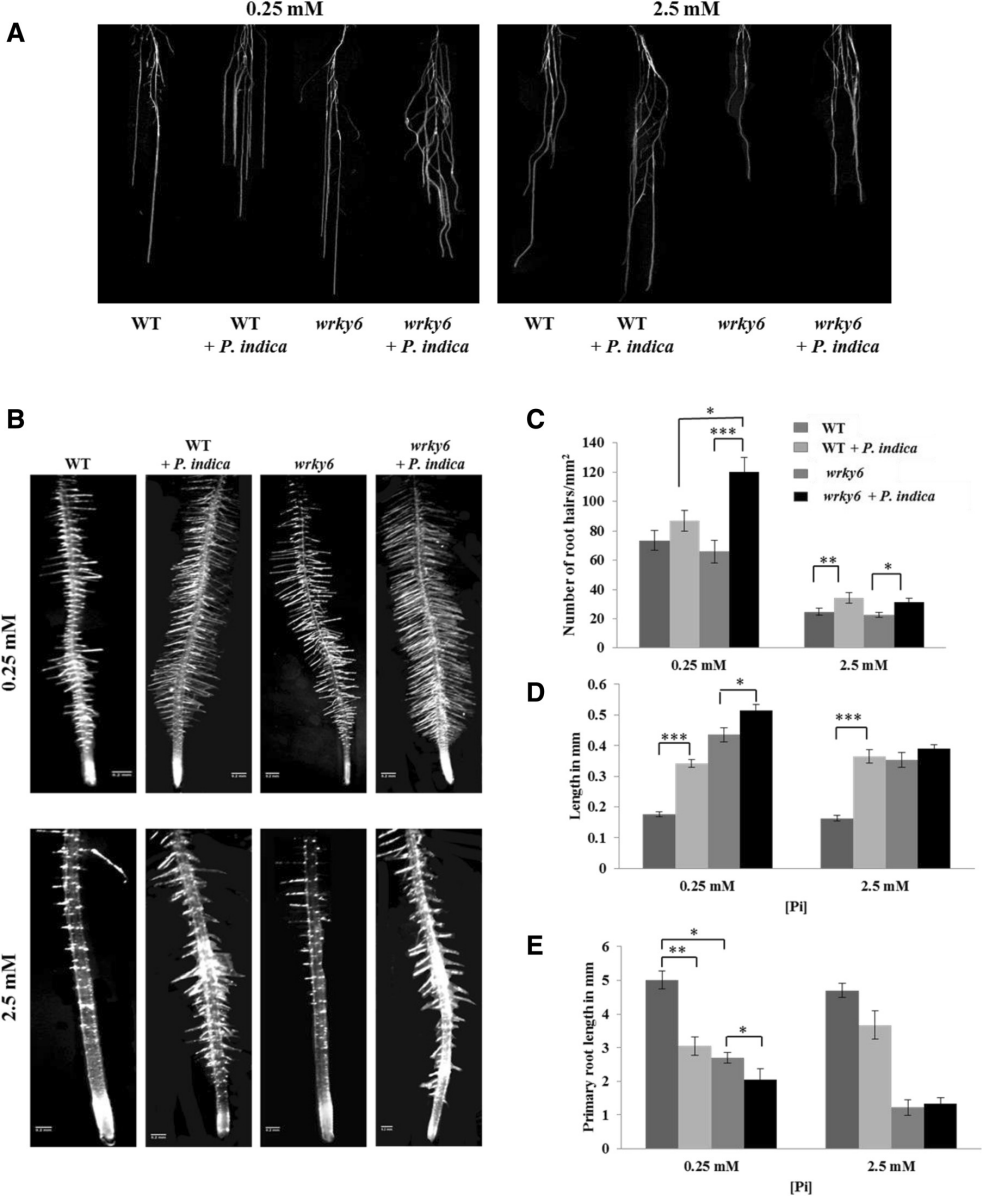
**a** Lateral root hairs of WT and *wrky6* seedlings grown under the two different Pi conditions with (+) or without (−) *P. indica* after 14 days on PNM medium. Photos are representative for more than 10 repetitions. Bar: 0.2 mm. **b** Root tips. **c** Root hair density, expressed as average number of root hairs/mm^2^, determined between 1 and 2 cm away from the root tip. **d** Average root hair lengths, determined for the same root section as in (**b**). **e** Primary root lengths after 14 days on PNM medium. Graphs are based on 3 independent experiments with 20 plants each. Bars represent SEs. Asterisks indicate significant differences, as determined by Student’s paired *t*-test for two tailed distribution (* *P* ≤ 0.05; ** *P* ≤ 0.01; *** *P* ≤ 0.001)

WT and *wrky6* seedlings (Fig. 1) were grown with/without *P. indica* on vertical PNM plates containing 2.5 or 0.25 mM Pi for 3, 6 and 12 days. A growth-promoting effect of the fungus on the seedling’s development became visible 3 days post incubation (dpi) and increased with decreasing Pi concentrations in the medium. In particular, root growth of both WT and *wrky6* seedlings was promoted with decreasing Pi concentrations and further stimulated by the fungus (Figs. 1 and 2). Closer inspection revealed that the roots are denser and bushier in the presence of *P. indica* because the number and lengths of both lateral roots (Figs. 1 and 2a) as well as root hairs (Fig. 2b–d) were increased. Furthermore, stimulation of root growth by *P. indica* in the WT under Pi limitation was restricted by WRKY6. This is particularly striking for seedlings grown on 0.25 mM Pi (Figs. 1 and 2): the growth-stimulating effect of *P. indica* is much stronger for *wrky6* roots than WT roots (Fig. 2b–d). Finally, consistent with the literature on root development after AM colonization [2], the primary root lengths of WT and *wrky6* seedlings were shorter under Pi limitation conditions (Fig. 2e). These results suggest that WRKY6 has a strong influence on the root architecture and that *wrky6* plants perform better than WT plants both in response to *P. indica* and Pi limitation.

**Fig. 3:**
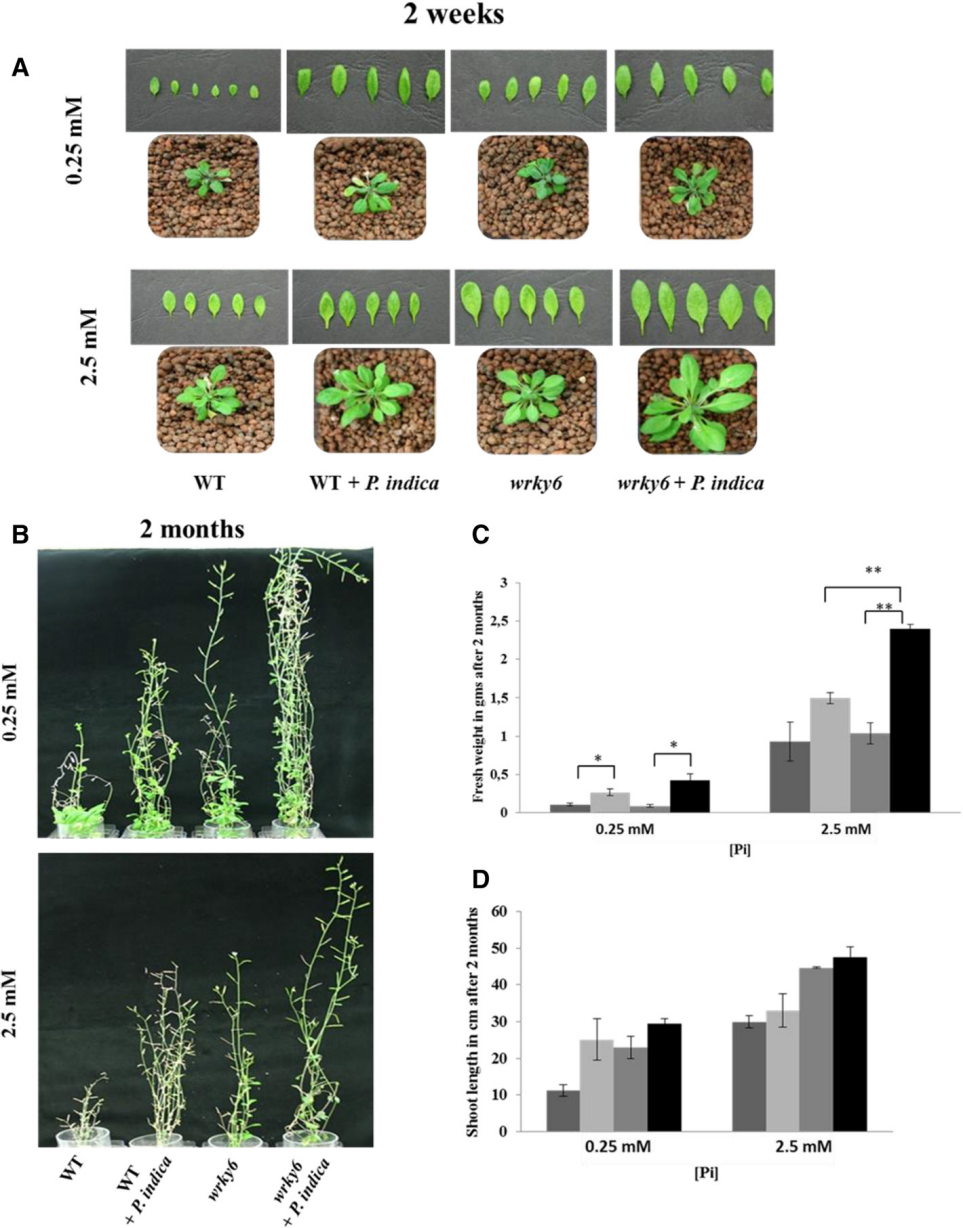
**a** Phenotypes of adult WT and *wrky6* plants grown in the absence or presence of *P. indica* on LP or NP. For growth conditions, cf. Methods and Results. **b** Typical view of Arabidopsis plants grown in expanded clay for 2 months. **c** Quantification of fresh weight in grams. **d** Quantification of shoot length in cm after long term interaction in expanded clay for 2 months. Data are based on 3 independent experiments with 5 plants each. Bars represent SEs. Asterisks indicate significant differences, as determined by Student’s paired *t*-test for two tailed distribution (** *P* ≤ 0.01; * *P* ≤ 0.05)

Phenotypic differences become more obvious after long term interaction of the symbionts in expanded clay. After 14 days of co-cultivation in Petri dishes, *P. indica*-colonized or mock-treated WT and *wrky6* seedlings grown on NP (2.5 mM, normal Pi) or LP (0.25 mM, low Pi) media were transferred to expanded clay. After 2 weeks, the first differences were observed in the size, shape and area of the leaves (Fig. 3a). *P. indica*-colonized *wrky6* seedlings were bigger under the two Pi concentrations, compared to seedlings on vertical agar plates. Although all plants showed Pi stress symptoms when grown under LP conditions for 2 months, the *wrky6* line performed better than the WT, and this was even more pronounced in the presence of *P. indica*. In particular, after 2 months, a significant increase in fresh weight (Fig. 3b, c) and shoot length (Fig. 3d) was observed for *P. indica*-treated *wrky6* seedlings (grown on NP medium) compared to the untreated control and the WT. This confirms that WRKY6 restricts *P. indica*-mediated growth promotion.

### Low Pi enhanced *P. indica* colonization in WT seedlings

**Fig. 4:**
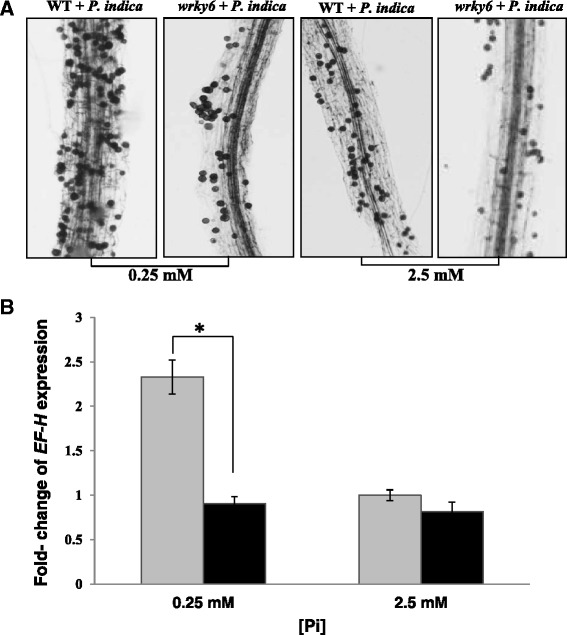
**a** Colonization of WT and *wrky*6 Arabidopsis roots by *P. indica* after co-cultivation under 0.25 mM and 2.5 mM Pi concentrations in the media. The fungal material was stained with Trypan Blue. Photos are representative for more than 10 repetitions. **b** Quantification of the degree of root colonization by quantitative RT-PCR. The amount of fungal material (determined as fungal DNA with *P. indica*-specific primers) is expressed relative to the plant *GAPDH* DNA (determined with *GAPDH*-specific primers). Graphs are based on 3 independent experiments with 20 plants each. Bars represent SEs. Asterisk indicates significant difference, as determined by Student’s paired *t*-test for two tailed distribution (* *P* ≤ 0.05)

To assess the effect of Pi on root colonization, the fungal spores associated with Arabidopsis roots were stained with Trypan Blue. Fig. 4a demonstrates that the number of spores associated with WT roots was higher under LP than NP conditions, and this was not observed for *wrky6* seedlings. The colonization by *P. indica* was also confirmed by quantitative RT-PCR with the *P. indica*-specific marker gene *EF- H*, relative to the plant *GAPDH* gene (Fig. 4b). These results highlight the strong effect of root colonization for WT seedlings grown under LP conditions (cf. Discussion).

### *P. indica* stimulates ethylene (ET) production in LP-grown *wrky6* seedlings

**Fig. 5:**
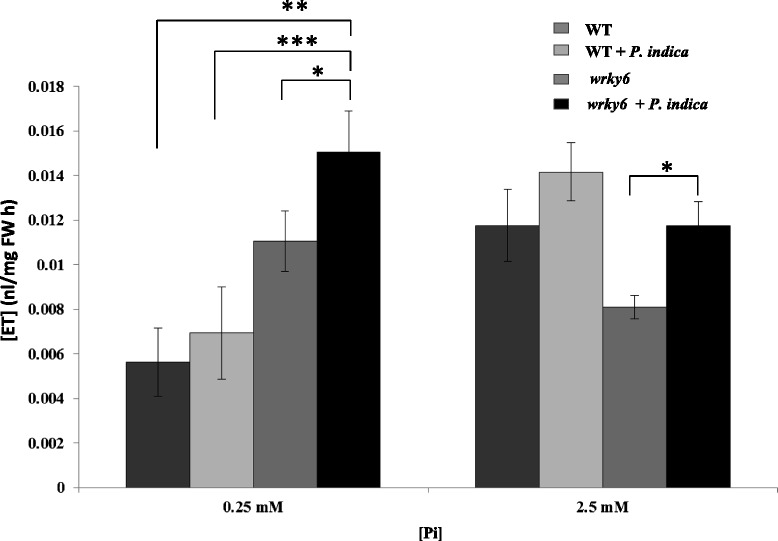
ET levels of WT and *wrky6* seedlings which were either co-cultivated with *P. indica* for 2 weeks or mock-treated. Data are based on 3 independent experiments with 20 plants each. Bars represent SEs. Asterisks indicate significant differences to the ET levels of seedlings grown under NP conditions, as determined by Student’s *t*-test (* *P* ≤ 0.05; ** *P* ≤ 0.01; *** *P* ≤ 0.001)

ET plays an important role in primary root growth and root hair formation in seedlings growing under Pi limitation [37], and has a strong influence on hyphal growth, branching and root colonization [38]. As shown in Fig. 5, ET released from WT seedlings was > 2-fold lower under LP than under NP conditions. The ET production of uncolonized *wrky6* seedlings was comparable under the two Pi concentrations. In all instances, *P. indica* stimulated ET production, but significant stimulation was only observed for *wrky6* seedlings. LP-, but not NP-grown mutant seedlings produced ~2-times more ET than the WT, irrespective of whether the seedlings were exposed to the fungus or not. The higher ET production of *wrky6* seedlings under LP conditions compared to the WT might contribute to the change in the root architecture and the lower root colonization of the mutant (37–38, cf. Discussion).

### *P. indica* promotes *PHOSPHATE1* (*PHO1*) expression under Pi limitation

**Fig. 6:**
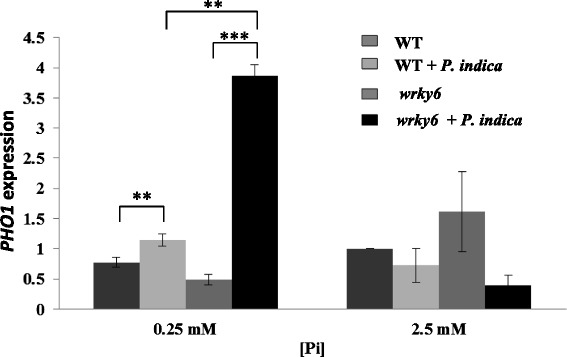
*PHO1* expression after 3 days of co-cultivation or mock-treatment of the two symbionts under the two different Pi concentrations. Data are based on 3 independent experiments with 15 plants each. Bars represent SEs. Asterisks indicate significant differences, as determined by Student’s paired *t*-test for two tailed distribution (** *P* ≤ 0.01; *** *P* ≤ 0.001)

PHO1 is a high affinity Pi transporter expressed predominantly in the roots, and the gene is up-regulated under low Pi conditions [39]. We observed a ~3.8-fold stimulation of *PHO1* expression by *P. indica* in *wrky6* roots under Pi limitation, but not under NP conditions (Fig. 6). This confirms previous observations that WRKY6 acts as a repressor of *PHO1* expression under NP conditions. The strong effect of *P. indica* on root development is reflected by the up-regulation of *PHO1* under Pi limitation.

### ^32^P uptake and inorganic Pi content

**Fig. 7:**
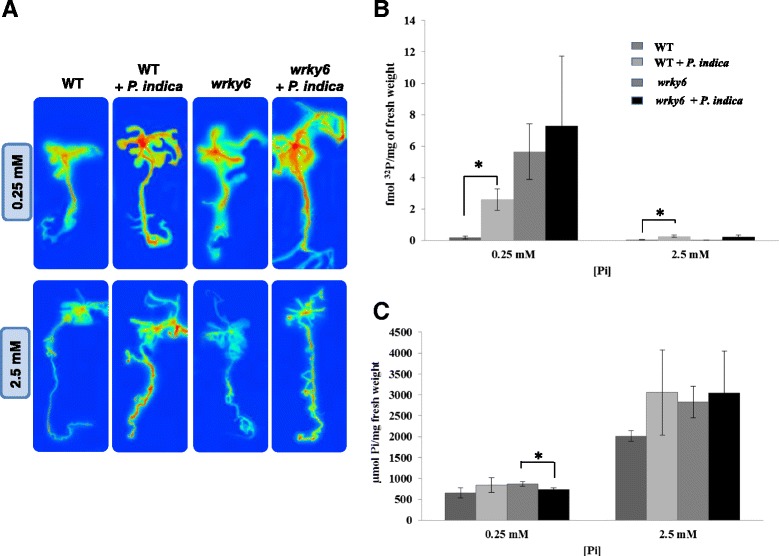
^32^P uptake and Pi content in WT and *wrky6* seedlings exposed to *P. indica* (or mock-treated) under NP and LP conditions. **a** ^32^P uptake. WT and *wrky6* mutants were co-cultivated with or without *P. indica* under the two Pi concentrations for 5 days before application of 2.5 μCi ^32^Pi. After additional 3 days, the radioactivity of the seedlings was visualized by autoradiography. False color presentations, whereas red represents high and blue low radioactivity. **b** Quantification of the data by liquid scintillation counting. The graph shows fmol of radioactive ^32^P/mg root fresh weights. **c** Inorganic Pi concentration/mg fresh weight after 14 days of co-cultivation (or mock treatment) of WT and *wrky6* seedlings with *P. indica*. Data are averages of 3 biological and 3 technical replicates. Bars represent SEs. Asterisks indicate significant differences, as determined by Student’s *t*-test (* *P* ≤ 0.05)

Figure 7a shows that the radioactivity in all parts of the *P. indica*-exposed seedlings is higher than in non-colonized plants, irrespective of whether they were grown under NP or LP conditions, and we observed a ~ 2-fold stimulation of ^32^P uptake in the presence of the fungus in both WT and *wrky6* seedlings (Fig. 7b). The comparable stimulation of Pi uptake by *P. indica* in the two genotypes demonstrates that the strong fungus-induced growth alteration in the *wrky6* mutant is not exclusively caused by a more efficient Pi uptake. In addition, the total amount of Pi in the seedlings cannot explain the fungus-induced phenotypic differences between *wrky6* and WT (Fig. 7c). As expected, the total Pi content in the seedlings is dependent on the Pi concentration in the medium, and seedlings grown under LP conditions contain less Pi than those grown on NP conditions. However, for a given Pi concentration in the medium, we did not observe significant differences of the total Pi content in the seedlings of the two genotypes or the presence or absence of the fungus (Fig. 7c). Thus, also the comparable amount of Pi in colonized and uncolonized WT and *wrky6* seedlings cannot explain the differences in the root architecture observed among the seedlings grown under LP conditions (cf. Discussion).

**Fig. 8:**
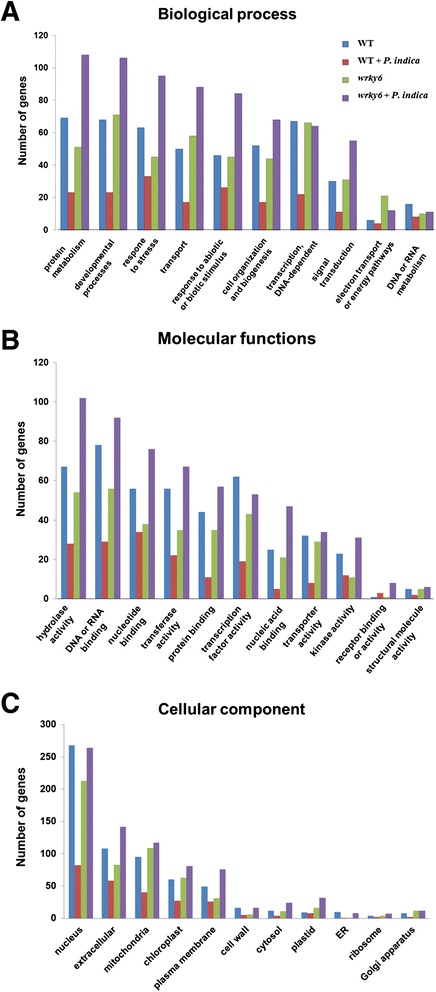
Functional categorization of genes which are regulated more than 2-fold in response to LP based on *A. thaliana* Gene Ontology (TAIR’s GO annotations). **a** Genes involved in “biological process”. **b** Genes involved in “molecular function”. **c** Genes involved in “cellular component”

### Pi-regulated genes in *P. indica*-colonized *wrky6* roots

The strongest stimulation of root growth was observed when NP-grown *P. indica*-colonized *wrky6* seedlings were compared to those grown on LP (Figs. 1 and 2). Therefore, we first identified genes which were regulated by *P. indica* only in *wrky6* roots (and not in the WT) and only under Pi limitation (Additional file 1: Table S1). Mapman categorization revealed that 9 auxin-related genes code for small auxin up RNAs (SAURs). Others code for an auxin efflux regulator, auxin response factors (e.g. ARF12), auxin-regulated TFs (e.g. LEAF COTYLEDON2) or are auxin targets (e.g. expansins, cell wall biosynthesis enzymes). Down-regulation of *IAA34*, encoding a repressor of ARFs [40, 41] further supports that the auxin metabolism is activated. Two genes ([*NINE-cis-EPOXYCAROTENOID DIOXYGENASE4* [42] and *HVA22* [43]) are involved in ABA functions. GA REQUIRING1, GA 20-OXIDASE3 and GA 2-OXIDASE7 are key players in gibberellin (GA) biosynthesis [44, 45].

Numerous transport processes are stimulated, as shown by the regulation of genes for p- and v-ATPases, carbohydrate, amino acid, lipid, nucleotide, Pi, nitrate and metal transporters, ABC and metabolite transporters, as well as aquaporins.

Re-organisation of the root architecture is also reflected by the stimulation of genes for the primary (e.g. glucose) and secondary (e.g. stress) metabolisms, developmental processes, cell organization, cell cycle, vesicle transport, growth regulators and early signaling compounds. Among the latter group are compounds (such as the Ca^2+^-binding CALMODULIN-LIKE37 and receptor kinases) which have not yet been analyzed in roots. This highlights that many of the *P. indica*-induced responses to LP stress in the WT are restricted by WRKY6.

Interestingly, 25 miRNAs, 10 with known and 15 with unknown functions, are regulated in the bigger roots. *miR156G*, *miR169F*, *miR395B* and *miR399C* respond to Pi starvation [46, 47]. *miR394B* targets the mRNA for an F-box protein of the SKP1-Cullin/CDC53-F-box complex, and is involved in auxin responses [48]. *miR169F* targets the mRNA for the subunit A of the NF-Y TF complex thereby controlling primary and lateral root initiation [49]. Furthermore, *ALKENYL HYDROXALKYL PRODUCING2* is involved in glucosinolate biosynthesis and predicted to be targeted by *miR826* and *miR5090*, and both miRNAs are induced in response to Pi starvation [50]. All these miRNAs are regulated by *P. indica* in LP-exposed *wrky6*, but not WT roots. This highlights the importance of this TF on the restriction of root development under Pi limitation.

A functional categorization of genes based on a) involvement in” biological process”, b) involvement in “molecular function”, c) involvement in “cellular component” and regulated more than 2-fold in response to LP based on A. thaliana Gene Ontology (TAIR’s GO annotations) is given in (Fig. 8).

### Proposed list of general genes involved in root growth promotion

**Fig. 9:**
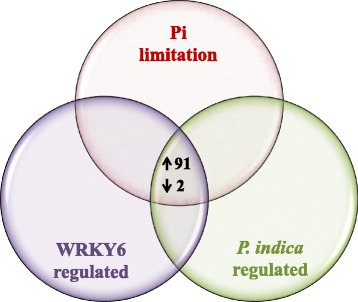
Common up- and down-regulated genes (>4 fold) in Arabidopsis roots regulated either by Pi limitation, WRKY6 or *P. indica* (Venn diagram). For experimental details, cf. Methods and Results

The Venn-diagrams (Fig. 9) generated by the MAPMAN software identified genes, which were regulated by LP (but not *P. indica* or WRKY6), by *P. indica* (but not LP or WRKY6), and by WRKY6 (but not *P. indica* or LP). The common genes among these three datasets are not specific for one of the three stimuli and should therefore represent more general genes involved in the promotion of root growth and development (Fig. 9). Those genes were then arranged according to their average fold regulation in all datasets (Table 1, 4-fold; Additional file 2: Table S2). The higher the genes are ranked in the list, the more important appears to be their requirement during root growth.

**Table 1:** Genes which are regulated more than 4-fold (log2 value ≥ 2) in response to Pi limitation, *P. indica* and mutation of *WRKY6*

Mapman BinCode	Gene ID	Gene Description	Pi limitation	*P. indica*	*wrky6*
20.2.3	At1g26850	dehydration-responsive family protein	6.0	7.1	6.9
35.1.26	At4g11540	DC1 domain-containing protein involved in intracellular signaling	5.9	7.0	6.9
35.2	At4g08593	unknown protein	6.4	6.3	6.9
27.3.72	At5g67480	BTB AND TAZ DOMAIN PROTEIN 4	5.8	6.9	6.8
35.2	At3g09975	unknown protein	6.6	6.7	5.9
35.2	At5g46220	unknown protein	5.9	5.8	5.6
27.3.71	At4g29160	SNF7	5.6	5.9	5.8
26.21	At4g33355	lipid binding protein	5.7	5.1	5.8
21.4	At4g08550	electron carrier/ protein disulfide oxidoreductase	5.5	5.4	5.1
35.2	At3g45880	2-oxoglutarate and Fe^2+^-dependent oxygenase	5.8	5.8	4.2
30.3	At1g32250	putative calmodulin	5.4	5.3	5.1
29.5	At5g09640	SCPL19 (serine-type carboxypeptidase)	4.8	5.6	4.8
35.1	At2g01790	meprin and TRAF homology domain-containing protein	5.1	5.1	5.0
27.3.41	At3g46770	transcription factor of the B3 family	2.2	6.4	6.4
27.3.11	At1g51220	WIP5 (zinc finger protein)	4.8	5.3	4.7
35.2	At2g18200	unknown protein	5.1	4.9	4.8
27.3.24	At1g65360	AGL23 (AGAMOUS-LIKE 23)	4.9	5.1	4.5
29.5.11.4.3.2	At5g44980	F-box family protein	6.6	3.5	4.4
27.3.67	At5g27140	putative SAR DNA-binding protein	3.0	5.9	5.4
27.2	At1g30455	transcription factor	4.6	5.1	4.5
33.99	At5g62850	SWEET5	4.7	4.7	4.7
35.2	At4g27930	unknown protein	4.5	4.7	4.7
17.2.3	At4g34780	auxin-responsive protein	4.8	4.0	4.9
27.4	At5g53720	RNA recognition motif -containing protein	4.4	4.5	4.5
29.4	At1g43895	unknown protein	4.2	4.5	4.5
27.3.99	At2g26135	zinc finger protein	4.6	4.0	4.6
10.8.1	At1g69940	PPME1 (pectinmethylesterase)	4.3	4.1	4.5
26.8	At1g01980	reticuline oxidase-like protein	4.2	4.3	4.3
35.2	At4g25990	CIL (chloroplast import apparatus 2-like protein)	4.4	4.4	3.8
35.1.12	At5g56510	APUM12 (Arabidopsis PUMILIO 12)	3.0	5.0	4.6
35.2	At3g43572	unknown protein	5.9	2.4	4.1
35.2	At3g59620	unknown protein	3.6	4.4	4.6
21.1	At2g33270	ACHT3 (atypical cysteine/histidine-rich thioredoxin 3)	4.7	3.6	4.0
20.1.7	At3g48231	LCR48 (low-molecular-weight cysteine-rich protein 48)	3.9	4.1	4.2
35.2	At3g50376	unknown protein	4.1	4.2	3.9
35.2	At1g55221	unknown protein	4.6	3.6	3.3
31.4	At1g07725	ATEXO70H6 (exocyst subunit EXO70 family protein H6)	3.7	3.9	3.5
35.2	At2g17305	unknown protein	3.8	3.9	3.4
35.2	At4g29200	beta-galactosidase	3.5	3.8	3.9
33.99	At5g07930	MCT2 (mei2 C-terminal RRM only like 2 protein)	4.2	2.8	4.2
35.2	At5g45690	unknown protein	4.0	4.2	2.9
35.1	At4g33820	glycosyl hydrolase family 10 protein	4.2	4.4	2.5
27.3.24	At5g51860	AGL72 (MADS-box protein)	3.9	4.0	3.2
27.3.7	At3g21880	zinc finger (B-box type) protein	3.5	3.7	3.8
35.2	At1g24256	unknown protein	2.4	3.9	4.6
35.2	At5g28295	unknown protein	3.6	3.4	3.5
35.1	At3g57840	self-incompatibility protein-related protein	3.1	3.7	3.6
20.1	At2g15040	ATRLP18 (receptor-like protein 18)	4.4	2.2	3.7
29.5.11.4.3.2	At5g53840	FBL13 (F-box family protein 13)	3.4	3.4	3.4
35.2	At2g11440	unknown protein	3.6	3.6	3.0
35.1	At3g48620	unknown protein	3.7	2.7	3.7
29.3.4.1	At2g38960	AERO2 (Arabidopsis endoplasmic reticulum oxidoreductins 2)	4.0	2.3	3.4
35.1	At4g26860	pyridoxal phosphate binding protein	3.7	2.9	3.1
29.5.11.4.3.2	At5g44220	F-box family protein	3.7	2.3	3.7
20.1.7	At4g09984	LCR34 (low-molecular-weight cysteine-rich protein 34)	3.2	3.3	3.2
27.1	At5g03580	Putative polyadenylate-binding protein	3.4	3.4	2.9
35.2	At4g08022	unknown protein	3.4	3.4	2.8
35.2	At4g05018	unknown protein	3.7	3.1	2.7
33.99	At1g21890	nodulin MtN21 family protein	2.9	3.1	3.5
35.2	At1g23910	unknown protein	3.0	3.6	2.9
24	At5g16080	CXE17 (carboxyesterase 17)	3.5	2.9	3.0
35.2	At5g51090	unknown protein	3.3	2.9	3.2
33.99	At2g30300	nodulin-related protein	3.2	3.0	3.0
33.99	At2g37860	LCD1 (LOWER CELL DENSITY 1)	3.0	3.3	2.9
26.4.1	At3g24330	glycosyl hydrolase 17	2.9	3.0	3.1
35.1	At3g48209	thionin family protein	3.0	3.0	2.8
20.1.7.12	At4g14272	defensin-like protein	3.1	2.9	2.7
10.8.1	At1g11590	putative pectin methylesterase	2.9	2.9	2.8
29.5.11.1	At5g48700	ubiquitin-related protein	2.5	3.4	2.6
35.2	At3g30520	unknown protein	2.8	2.8	2.8
11.1.8	At1g21540	AMP-dependent synthetase and ligase family protein	3.0	2.7	2.4
35.2	At3g43829	unknown protein	4.0	2.1	2.0
35.2	At5g29044	unknown protein	3.8	2.2	2.1
35.1	At3g58290	meprin and TRAF homology domain-containing protein	2.7	3.0	2.4
35.2	At1g57906	unknown protein	2.7	2.6	2.8
26.3.2	At4g38590	glycosyl hydrolase 35	2.9	2.4	2.5
30.2.9	At1g24650	leucine-rich repeat family protein	2.3	2.6	2.9
27.3.24	At2g24840	AGL61 (AGAMOUS-LIKE 61)	2.6	2.8	2.3
35.1.41	At1g30795	hydroxyproline-rich glycoprotein family protein	2.9	2.4	2.3
29.5.7	At3g59990	MAP2B (METHIONINE AMINOPEPTIDASE 2B)	2.6	2.4	2.7
16.2	At1g32910	transferase	2.8	2.0	2.6
27.3.67	At1g61320	unknown protein	2.4	2.2	2.8
35.2	At5g50360	unknown protein	2.1	2.5	2.8
35.2	At1g03240	unknown protein	2.5	2.5	2.3
35.1	At4g19910	Toll-Interleukin-Resistance (TIR) domain-containing protein	2.8	2.3	2.2
35.1	At3g06880	nucleotide binding protein	3.0	2.1	2.2
35.1	At5g52690	heavy-metal-associated domain-containing protein	2.4	2.4	2.2
26.10	At1g19630	CYP722A1 (monooxygenase)	2.5	2.5	2.0
35.2	At3g58300	unknown protein	2.3	2.3	2.3
35.2	At3g43950	phosphotransferase	2.2	2.3	2.1
35.2	At1g53285	unknown protein	2.3	2.3	1.8
35.2	At4g20520	RNA binding/RNA-directed DNA polymerase	−2.5	−2.3	−2.5
17.1.1	At2g36020	HVA22J (HVA22-LIKE PROTEIN J)	−4.2	−7.1	−2.2

91 genes are regulated more than 4-fold (log_2_ > 2) in all 3 datasets and only 2 of them are down-regulated (Table 1). For 28 of them, we could not find sufficient information to predict a function of their products. In addition, for 59 gene products, we did not find functional analysis data or predictions for roots.

It appears that root growth is associated with water shortage (At1g26850) and a high demand for sugar (At5g62850). SWEET5 appears to play a major role in providing photoassimilates *via* the phloem to the roots and for the fungus, as its message is the only one of the SWEET sucrose efflux transporter gene family [51] in the list. The auxin/cytokinin ratio is important for root/shoot ratios. Only 3 genes involved in the auxin and cytokinin metabolism are in the list: SAUR2 participating in cell expansion, an auxin-inducible uncharacterized leucine-rich repeat protein (At1g24650) [52] and the predicted TF At5g27140 which responds to cytokinin through the histidine-to-aspartate photorelay circuit [53]. Interestingly, no other hormone-related genes were highly ranked among the common genes.

Among the proteins known to be involved in cell wall extension such as xyloglucan endotransglucosylase-hydrolases, expansins, polygalacturonases or peroxidases, the uncharacterized pectin methylesterases At1g69940 and At1g11590 appear to be important. Cell growth requires an increase in exocytosis, which is reflected by the highly ranked *SNF7* gene in the list. SNF7 is involved in internal vesicle formation of the prevascular compartment [54]. Specific members of gene families (such as At1g07725 of the EXOCYST70 family) participate in the stimulation of the export in growing cells. The increased demand for lipids is reflected by genes involved in lipid metabolism (At4g33355; At1g21540). Control of the redox potential (At3g45880, At2g33270) and specific plastid functions, such as the import into the organelle (At5g07930), appear to be important. Rapid responses to developmental changes have been associated with posttranscriptional processes mediated by PUMILIO proteins (At5g56510) [55]. Closer inspection of the genes regulated > 4-fold using the TAIR and NCBI databases revealed that additional not well characterized proteins are potentially associated with root or cell growth, but this requires further studies. In addition, extension of the list of genes by reducing the threshold level to 2-fold regulation in all three conditions (Additional file 2: Table S2, 2-fold) uncovered additional ~ 100 proteins with predicted growth related functions (TAIR homepage), such as REPRODUCTIVE MERISTEM1, GROWTH REGULATING FACTOR4, EXPANSIN23, the RmIC-like cupin protein At1g03890, HISTONE ACETYLASE18, the Ca^2+^-dependent PROTEIN KINASE14, LIFEGUARD1 and SYNAPTOTAGMIN2, to mention a few. Finally, the function of the strongly down-regulated HVA22J-like PROTEINJ needs to be analyzed.

In summary, very limited information is available for the majority of the genes which respond to the three root growth stimulators analyzed in this study.

## Discussion

Low Pi is a major stress for plants. Therefore, plants have evolved complex mechanisms for acquisition, re-mobilization and recycling of Pi to maintain the P homeostasis in a cell. Spatio-temporal molecular, physiological and biochemical Pi deficiency responses are the consequence of local and systemic sensing and the activation of signaling pathways. They stimulate Pi metabolism, but also initiate developmental reprogramming leading to changes in the root system architecture [56]. Among the 74 WRKY members in Arabidopsis, WRKY6, −42 and −75 are involved in LP stress [34, 35]. For the studies performed here, we observed a strong positive effect on plant performance when the *WRKY6* gene was inactivated. Therefore this WRKY protein was investigated in more details. Furthermore, *P. indica* helps plants to adapt to various stress conditions and supplies them with nutrients including Pi which leads to root growth promotion [14, 16]. Interestingly, Müller et al. [57] performed microarray analyses for Pi-starved Arabidopsis leaves. 73 of their genes are also regulated in our microarrays with WT roots. The number is reduced to 37 when the seedlings are grown in the presence of *P. indica* (compare Müller et al. [57]). This confirms that the fungus reduces the Pi stress response in Arabidopsis roots. Here, we demonstrate that the three unrelated factors “presence of *P. indica*”, “limitation of Pi” and “absence of WRKY6” strongly influence plant growth and in particular the root architecture. The stimulating effects of the fungus and Pi limitation are restricted by WRKY6. Under NP conditions, the difference in the response to *P. indica* among the two genotypes is smaller than under LP conditions (Figs. 1 and 2). These effects are not only visible at the seedling’s level but persist after shifting the seedlings to expanded clay (Fig. 3). The root hair density and length are strongly promoted by *P. indica* in the *wrky*6 mutant in comparison to the WT grown under LP conditions (Fig. 2b–d), while the length of the primary roots is reduced (Fig. 2e). Chen et al. [34] also observed phenotype differences between WT and *wrky6* plants under LP conditions, but the root architecture was not analyzed in details. Robatzek and Somssich [58] did not observe differences between *wrky6* and WT, but their growth conditions were quite different from ours and those of Chen et al. [34]. Similar alterations in the root architecture in response to LP occur after AM colonization [59–61], and in *P. indica*-colonized Chinese cabbage seedlings [16, 62, 63]. Chinese cabbage showed a stronger response to *P. indica* than Arabidopsis. The interaction results in a bushy root phenotype, comparable to our observations with LP-grown *wrky*6 seedlings (Fig. 2).

PHO1 participates in the transfer of Pi from root epidermal and cortical cells to the xylem [39]. This may explain the better performance of the aerial parts of the *P. indica*-colonized mutant under Pi limitation. Low Pi treatment reduced WRKY6 binding to the *PHO1* promoter [34]. Furthermore, inactivation of *WRKY6* stimulated *PHO1* expression by *P. indica* under LP, but not NP conditions (Fig. 6). This suggests that a WRKY6-independent regulatory mechanism exists that stimulates *PHO1* expression under LP by signals from *P. indica*. Mycorrhizal symbiosis also enhances the expression of various Pi transporter genes like *OsPT11* in rice [64] and *MtPT4* in *Medicago trunculata* [65]. *P. indica* also stimulates the expression of other Pi transporter genes including *Pht1;5* in LP-grown *wrky6* seedlings. The *Pht1;5* promoter contains W-boxes [34, 35] which are putative binding sites for WRKY TFs. Since *Pht1;5* is not up-regulated in WT seedlings under these conditions, WRKY6 might function as a transcriptional repressor for this gene (data not shown). Furthermore, consistent with previous observations [18, 19, 66], we found an increase in Pi uptake in the presence of *P. indica* under low Pi conditions, however there is no difference between WT and *wrky6* seedlings (Fig. 7). Stimulation of Pi uptake by *P. indica* might establish local Pi gradients which could result in altered local Pi stress responses, and a reprogramming of root developmental programs. These programs are initiated by Pi limitation and further promoted by *P. indica* and the absence of WRKY6. Mycorrhizal fungi association is a well-known strategy of plants for enhancing Pi uptake [61, 67]. In spite of a more efficient Pi uptake in the presence of *P. indica*, the overall Pi content in colonized or uncolonized WT and *wrky6* seedlings is not different, although seedlings grown under Pi limitation conditions contain less Pi than those grown under NP conditions (Fig. 7). This again is consistent with the idea that limitations in the Pi availability induce growth and alterations in the root architecture. *P. indica* either reduces the Pi limitations or interferes with the signaling events activating the Pi stress response. Plants grown under Pi limitation use their own Pi reservoir to maintain Pi homeostasis within cells [68] and simultaneously stimulate the Pi uptake machinery [69, 70]. The RmIC-like cupins protein which is up-regulated at the mRNA level during root growth (Additional file 1: Table S1, Additional file 2: Table S2) has been proposed to have nutrient reservoir activity and is a candidate for controlling Pi availability.

In LP conditions, ET is an important factor for inhibition of primary root growth and promotion of lateral root elongation [2, 37]. Plant-derived ET also stimulates spore germination and hyphal growth of vesicular AM [38, 71]. As shown in Fig. 5, colonized and un-colonized mutant seedlings produced ~2-times more ET than WT seedlings. Since WT plants with less ET production are more colonized than *wrky6* plants irrespective of the Pi level in the root environment (Fig. 4), ET may restrict root colonization. Thus, this hormone might be important to balance growth of the microbe, the resulting benefits for the host, and the degree of defense gene activation of the host against the invader. Under our growth conditions, the higher ET level in *wrky6* plants may stimulate lateral root development, spore germination and hyphal growth, which is consistent with the better performance of the *wrky6* roots. The important role of ET and ET signaling components for mutualistic interaction of Sebacinales with various plant species has also been demonstrated by Khatabi and Schäfer [30], Camehl et al. [72] and Barazani et al. [23]. Taken together, WRKY6 is a crucial player in controlling root development in response to *P. indica* and Pi limitation.

### Microarray analyses

Since the strongest growth-promoting effect and change in the root morphology were observed for *P. indica*-colonized *wrky6* seedlings in LP, we used the roots of these seedlings to identify genes which cause this phenotype (Fig. 2). Many genes with known growth-related functions were identified (Additional file 1: Table S1, Additional file 2: Table S2). This includes genes for proteins involved in the primary metabolism (e.g. for the generation of energy-rich components), cell wall metabolism (e.g. methyl-pectinerases, expansins), hormone biosynthesis and signaling (proteins involved in auxin-, gibberellin-, ET-, jasmonic acid-, brassinosteroid and strigolactone-associated processes), or secondary metabolism (e.g. for stress or defense compounds or antioxidants).

Various well-characterized genes for developmental processes were specifically up-regulated in these roots, such as genes for the GROWTH REGULATOR4 (At3g52910), for cell regulation, transporters of ions, peptides, oligonucleotides or other small molecules. Also previously described proteins involved in the *P. indica*/Arabidopsis interaction were detected: e.g. components involved in protein sorting (cf. [73]), cytoskeleton rearrangement (cf. [21]) and MATH domain-containing proteins [74]. However, the majority of the genes are not or little characterized or not studied in roots yet.

WRKY6 restricts auxin-mediated growth responses in the WT (cf. also [75]). Among the identified genes is SAUR21 which contributes to cell expansion and basipetal auxin transport [76]. SAUR1, −6, −7, −17, −27, −49, −64 and −65 participate in various aspects of root development (TAIR homepage). The AUXIN-RESPONSE FACTOR (ARF)12 functions in Pi homeostasis in rice [77] and is essential for root growth through maintaining a correct polarization of the auxin transport machinery in Arabidopsis [78]. Expansin 10, B1, B3 and A5 participate in cell expansion and root epidermis cell differentiation [79, 80]. Only four genes for enzymes involved in cell wall biosynthesis are in the list: cellulose synthase-like D4 involved in root cell tip growth [81], cellulose synthase 10 and cellulose synthase-like D6 and -G3. Yang et al. [82] demonstrated that jasmonate prioritizes defense over growth by interfering with the GA signaling cascade. Consistent with this idea we observe up-regulation of 3 key enzyme genes for GA biosynthesis, while relatively few genes involved in defense responses are up-regulated and many of them are even down-regulated in *P. indica*-colonized *wrky6* seedlings in LP (Additional file 1: Table S1).

The role of miRNAs in plant-microbe symbiosis with nitrogen-fixing rhizobia [83], AM fungi [16, 84, 85] and plants grown under biotic and abiotic stress [86, 87] is well documented. Several mi/siRNAs target members of the ARFs family are involved in auxin homeostasis and signaling supposing their participation in crucial stages of root development [88]. Ye et al. [16] reported *P. indica*-mediated induction of several miRNAs which ultimately leads to growth promotion and vigorous root development in Oncidium hybrid orchid. Consistent with a huge number of publications, it appears that control of root development involves miRNAs with various functions and targets (Li and Zhang [89]). It is particularly interesting to understand the function of the newly identified miRNAs regulated during root growth promotion. Taken together, the combination of these genes appear to be crucial for reprogramming root development under Pi limitation and the presence of *P. indica* in *wrky*6.

### General genes

Genes which are regulated by all three quite diverse growth stimuli should code for common components involved in root growth. Genes with higher priority for growth should show a stronger regulation in response to the three stimuli than those which are less required. Table 1 and Additional file 2: Table S2 propose genes for quite diverse functions, but they may highlight those cellular and molecular processes which need to be activated to promote root growth and development. Interestingly, a literature and database survey uncovered that for the majority of the gene products, very limited or no information is available for their role in root growth regulation. Among the strongest up-regulated genes is the well-studied *SNF7*, which codes for an interacting protein of the endosomal sorting complex required for transport (ESCRT)-III subunits. It regulates the formation of intraluminal vesicles of the prevacuolar compartments [54]. Another strongly regulated gene is *SWEET5*, a member of the sucrose phloem transporter family [51]. The specific role of SWEET5 in this scenario is unknown. Furthermore, it is interesting to note that only specific members of multigene families or a gene for one particular protein of a multiprotein complex respond to all three stimuli. The list of genes uncovered metabolic and signaling pathways which are limiting for root growth promotion. It is reasonable to assume that specific combinations of these genes/gene products are important, which can now be tested experimentally.

## Conclusion

We conclude that three unrelated factors “presence of *P. indica*”, “limitation of Pi” and “absence of WRKY6” influence *A. thaliana* growth and in particular the root architecture and propose that common genes which respond to all three growth stimuli are central for the control of root growth and architecture. These genes can be tested for optimizing root growth in model and agricultural plants.

## Methods

### Growth conditions of plant and fungus

*Arabidopsis thaliana* WT and *wrky6* seeds were surface sterilized and placed on Petri dishes containing MS [90] nutrient medium. After cold treatment for 48 h at 4 °C, the plates were incubated for 10 days at 22 °C under continuous illumination (100 μmol m^−2^ s^−1^). *P. indica* was cultured as described previously on Aspergillus minimal medium [91].

### Generation of the homozygous *wrky6* lines

Homozygocity of the SALK_012997 (N661529; European Arabidopsis Stock Centre) line was confirmed by PCR using a combination of a T-DNA left border primer and a gene-specific *WRKY6* right border primer (Additional file 1: Table S1). Two different T-DNA left border primers, LBa1 (TGGTTCACGTAGTGGGCCATCG) and LBa1.3 (ATTTTGCCGATTTCGGAAC) were used. An additional PCR was performed to identify homozygous seedlings for the insertions using the gene-specific primers LP and RP (Additional file 2: Table S3).

### Co-cultivation experiments

Co-cultivation of *A. thaliana* (WT and *wrky6*) with the fungus *P. indica* was performed under *in vitro* culture conditions on a nylon membrane placed on top of solified PNM media [91]. Square Petri dishes were divided into two equal parts and one *P. indica* disk was placed on each part and was grown for 10 days. After 48 h of cold treatment and 10 days of growth as described above, seedlings of equal sizes were used for the co-cultivation assays. For Pi stress treatment PNM media with two different Pi concentrations [2.5 mM (normal Pi - NP) and 0.25 mM (low Pi - LP)] were used. For each Pi concentration, 4 treatments were compared: WT, WT + *P. indica*, *wrky6* and *wrky6* + *P. indica*. Seedlings were maintained under two different Pi concentrations as mentioned above for 3, 5, 6, 12 or 14 days at 22 °C and 70–80 % humidity in a 16-h light/8-h dark cycle. Roots and shoots were harvested separately and frozen in liquid nitrogen for further analyses. Only roots were used for gene expression analyses. Kaefer media (KM) disks were used for mock treatment. Mock-treated seedlings grown on 2.5 mM NP were used as control.

### Experiments on expanded clay

After co-cultivation with *P. indica* or mock treatment for 14 days on PNM plates, seedlings were transferred to Magenta boxes containing autoclaved expanded clay (one plant per box). Seedlings were supplied with 30 ml liquid PNM media containing the two different Pi concentrations, once a week. Plants were grown in a temperature (22 °C) and moisture-controlled room with light from the top (80 ± 10 μmol m^−2^ s^−1^) under short-day conditions (8 h light and 16 h darkness). The light intensity was monitored weekly. The sizes of the seedlings were also monitored weekly and quantified after photography.

### Quantitative Real-Time-PCR

RNA was isolated from root tissues after 3 days post incubation (dpi) as described by Sun et al. [92]. All reactions were performed from three biological and three technical replicates. The mRNA levels for each cDNA probe were normalized with respect to the plant glyceraldehyde-3-Pi dehydrogenase (*GAPDH*) mRNA levels, which has been validated as a reference gene for roots inoculated with *P. indica* ([92], and references therein). Fold-induction values of target genes were calculated with the ΔΔCP equation of Pfaffl [93] and related to the mRNA level of target genes for mock-treated roots from NP, which were defined as 1.0. Primer pairs used in this study are given in Additional file 2: Table S3.

### Root colonization

Roots from plates were harvested after 14 dpi of co-cultivation and were washed intensively with distilled water before RNA extraction. *P. indica* was monitored with a primer pair for the *ELONGATION FACTOR1* (*PiEF-H*) mRNA. The mRNA levels for *PiEF-H* were normalized with respect to the plant *GAPDH* mRNA levels. Staining of hyphae and spores was performed with Trypan blue (0.05 %) prior to light microscopy [91].

### Pi content analysis

Seedlings were grown under the two different Pi conditions for 12 dpi as described above. Shoots and roots were sampled separately. Fresh mass were measured, before the samples were dried in an oven at 105 °C overnight. For Pi content analyses, samples were mixed with 2 ml of 65 % HNO_3_ and kept for one hour at 160 °C. The final volume was adjusted to 10 ml and the pH to 3.0–4.0. Finally, samples were mixed with ascorbic acid reagent and ammonium molybdate reagent (DIN 38405) and the Pi content was analyzed by the phosphomolybdenum blue reaction using the UV-160A spectrophotometer. Total Pi concentration was expressed in μmol/g dry weight. Experiments were repeated 3 times with independent material.

### ^32^P uptake assay

WT and *wrky6* mutant were co-cultivated with/without *P. indica* on PNM media with two different Pi concentrations as described above for 5 days. After 5 dpi, 2.5 μCi or 25 nM of ^32^P-ortho-Pi were added to each plant (1 plant per plate), and the seedlings were again allowed to grow for 3 days. Roots and shoots were harvested separately and washed several times in Na-citrate buffer (10 mM, pH 6.0). Roots and shoots were dried in an oven at 70 °C, weighted and digested with a tissue solubilizer (Rotiszint®-eco plus). The radioactivity was determined by liquid scintillation counting (LS 6500) using standard full channel programs in single isotope experiments.

### Determination of root hair density, length and primary root length

Arabidopsis seedlings were grown on square Petri dishes and kept vertically. Co-cultivation with *P. indica* was performed as described above with a few modifications: (a) for PNM media gelrite was used instead of agar, (b) no membranes were used to enhance the visibility of the roots. After 14 dpi the images of roots were taken under a stereomicroscope (Leica MZ6) and the digital images were traced by hand using ImageJ 1.47v (NIH). Finally the pixels were converted into the appropriate metric equivalents. For the determinations of primary root lengths, seedlings were grown on liquid medium with *P. indica* spores under the different Pi conditions for 14 days, stained with trypan blue for 5 min and then placed on glass slides. Pictures of seedlings were scanned using a Desktop scanner at 600 dpi. These scanned pictures were further analyzed using ImageJ 1.47v (NIH).

### Microarray analyses

Total RNA from roots of colonized WT and *wrky6* mutants from 3 independent biological experiments grown under NP and LP conditions were harvested at 3 dpi. RNA from roots of mock-treated WT and *wrky6* mutants were used as control. For each treatment, same amounts of RNA from three independent biological replicates were labeled and hybridized according to Agilent's One-Color Microarray-Based Gene Expression Analysis (OAK Lab GmBH, Hennigdorf, Germany). Quality of RNA samples were checked by photometrical measurements with the Nanodrop 2000 spectrophotometer (Thermo Scientific) and then analyzed on agarose gels (2 %) as well as by using the 2100 Bioanalyser (Agilent Technologies, CA) for determining the RNA integrity and the exclusion of potential contaminants. After verifying the quality of RNA, the Low Input Quick Amp Labeling Kit (Agilent Technologies) was used for generation of fluorescent complementary RNA (cRNA). Default cRNAs were amplified by using oligo-dT primers labeled with cyanine 3-CTP (Cye-3) according to the manufacturer’s protocol. Cye-3-labeled probes were hybridized to 8 × 60 k custom-designed Agilent microarray chips. For hybridization the Gene Expression Hybridization Kit (Agilent Technologies) was used. The hybridized slides were washed and scanned using the SureScan Microarray Scanner (Agilent Technologies) at a resolution of 3 μm generating a 20 bit TIFF file, respectively.

### Microarray data analysis

Data extractions from Images were performed using the Agilent’s Feature Extraction software version 11. Feature extracted data were analyzed using the DirectArray Version 2.1 software from Agilent. Normalization of the data was performed with DirectArray using the ranked median quantiles according to Bolstad et al. [94]. To identify significantly differentially expressed genes log_2_-fold changes are calculated and Student’s *t*- test was performed. In summary, raw data were normalized by rank median quantiles, intensity values from replicate probes were averaged, log_2_-ratios between the treatments were calculated and Student’s *t*-statistics applied to test for significance. Genes with log_2_-fold change < −1 or > 1 and *p*-value < 0.05 were considered to be significantly different. Genes were classified based on functional categories and pathways using the MapMan (http://mapman.gabipd.org/web/guest/ mapman) and *A. thaliana* Gene Ontology softwares (TAIR’s GO annotations) [95].

Microarray data were verified by qRT-PCR as described previously from three independent biological experiments with three technical replicates (Additional file 2: Table S4). The microarray data have been submitted to NCBI (GEO) under the accession number GSE63500 (https://www.ncbi.nlm.nih.gov/geo/query/acc.cgi?acc=GSE63500).

### Quantification of ET

For ET measurements, 100 mg shoot material from each treatment was collected into 4 ml vials (Roth, Germany). After 3 h ET accumulation, the measurement was performed with the ETD-300 ET detector (Sensor Sense B.V., Nijmegen, The Netherlands) as described in Bhattacharya and Baldwin [96] and Sun et al. [92].

### Statistical analyses

The statistical analyses for the microarray data have been described above. All additional statistical analyses were performed using Excel (2010) for Student’s paired *t*-test for two tailed distribution.

### Availability of supporting data

The data sets supporting the results of this article are included within the article and its additional files. The microarray data have been submitted to NCBI (GEO) under the accession number GSE63500.

## Additional files

Additional file 1: Table S1. Genes which are up-regulated more than 2-fold in *wrky6* + *P. indica* in LP (log_2_ value ≥ 1 or ≤ −1). (XLS 200 kb) Additional file 2: Table S2. Genes which were regulated more than 2-fold (log2 value ≥ 1) in response to Pi limitation, *P. indica* and mutation of *WRKY6*. **Table S3.** Primers used in this study. **Table S4.** Microarray validation with Real-time PCR. (PDF 563 kb)

## Abbreviations

*Pi*: *Piriformospora indica*
P: phosphorus
Pi: phosphate
AM: arbuscular mycorrhiza
TFs: transcription factors
PHO1: phosphate1
NP: normal phosphate
LP: low phosphate
dpi: days post incubation
ET: ethylene
WT: wild-type
