# Supplementary material for: WRKY6 restricts Piriformospora indica-stimulated and phosphate-induced root development in Arabidopsis
Source: BMC Plant Biol. 2015 Dec 30;15:305. doi: 10.1186/s12870-015-0673-4 (PMC4697310; doi:10.1186/s12870-015-0673-4)
Supplement: Additional file 2: Table S2. — Genes which were regulated more than 2-fold (log2 value ≥ 1) in response to Pi limitation, P. indica and mutation of WRKY6. Table S3. Primers used in this study. Table S4. Microarray validation with Real-time PCR. (PDF 563 kb) [file 12870_2015_673_MOESM2_ESM.pdf]

WRKY6 restricts *Piriformospora indica*-stimulated root development in Arabidopsis under phosphate limitation

Identification of genes that optimize root growth and development

Madhunita Bakshi[1], Khabat Vahabi[1], Samik Bhattacharya[2], Irena Sherameti[1], Ajit Varma[3], Kai-Wun Yeh[4], Ian Baldwin[2], Atul Kumar Johri[5], and Ralf Oelmüller[1*]

[1]Institute of General Botany and Plant Physiology, Friedrich-Schiller-University Jena, Dornburgerstr. 159, D-07743 Jena, Germany

[2]Max-Planck-Institute for Chemical Ecology, Beutenberg Campus, Hans-Knöll-Straße 8, D-07745 Jena, Germany

[3]Amity Institute of Microbial Technology; AUUP; Noida, India

[4]Institute of Plant Biology, Taiwan National University, Taipei, Taiwan

[5]School of Life Sciences; Jawaharlal Nehru University, New Delhi-110067, India

Additional file_2

Table S2. Genes which were regulated more than 2-fold (log2 - value ≥ 1) in response to Pi limitation, *P. indica* and mutation of *WRKY6*.

Mapman BinCode	Gene ID	Gene Description	Pi limitation	*P. indica*	*wrky6*
20.2.3	At1g26850	dehydration-responsive family protein	6.0	7.1	6.9
35.1.26	At4g11540	DC1 domain-containing protein, intracellular signaling	5.9	7.0	6.9
35.2	At4g08593	unknown protein	6.4	6.3	6.9
27.3.72	At5g67480	BT4 (BTB AND TAZ DOMAIN PROTEIN 4); transcription regulator	5.8	6.9	6.8
35.2	At3g09975	unknown protein	6.6	6.7	5.9
35.2	At5g46220	unknown protein	5.9	5.8	5.6
27.3.71	At4g29160	SNF7.1	5.6	5.9	5.8
26.21	At4g33355	lipid binding protein	5.7	5.1	5.8
21.4	At4g08550	electron carrier/ protein disulfide oxidoreductase	5.5	5.4	5.1
35.2	At3g45880	2-oxoglutarate (2OG) and Fe(II)-dependent oxygenase superfamily protein	5.8	5.8	4.2
30.3	At1g32250	calmodulin, putative	5.4	5.3	5.1
29.5	At5g09640	SCPL19; serine-type carboxypeptidase / sinapoyltransferase	4.8	5.6	4.8
35.1	At2g01790	meprin and TRAF homology domain-containing protein	5.1	5.1	5.0
27.3.41	At3g46770	transcriptional factor B3 family protein	2.2	6.4	6.4
27.3.11	At1g51220	zinc finger (C2H2 type) protein (WIP5)	4.8	5.3	4.7
35.2	At2g18200	unknown protein	5.1	4.9	4.8
27.3.24	At1g65360	AGL23 (AGAMOUS-LIKE 23); transcription factor	4.9	5.1	4.5
29.5.11.4.3.2	At5g44980	F-box family protein	6.6	3.5	4.4
27.3.67	At5g27140	SAR DNA-binding protein, putative	3.0	5.9	5.4
27.2	At1g30455	transcription factor	4.6	5.1	4.5
33.99	At5g62850	AtVEX1 (VEGETATIVE CELL EXPRESSED1), SWEET5	4.7	4.7	4.7
35.2	At4g27930	unknown protein	4.5	4.7	4.7
17.2.3	At4g34780	auxin-responsive family protein	4.8	4.0	4.9
27.4	At5g53720	RNA recognition motif (RRM)-containing protein	4.4	4.5	4.5
29.4	At1g43895	pseudogene, protein kinase -related protein	4.2	4.5	4.5
27.3.99	At2g26135	zinc finger (C3HC4-type RING finger) family protein	4.6	4.0	4.6
10.8.1	At1g69940	PPME1; pectinesterase	4.3	4.1	4.5
26.8	At1g01980	SEC1A	4.2	4.3	4.3
35.2	At4g25990	CIL, chloroplast import apparatus CIA2-like protein	4.4	4.4	3.8

35.1.12	At5g56510	APUM12 (ARABIDOPSIS PUMILIO 12); RNA binding protein	3.0	5.0	4.6
35.2	At3g43572	unknown protein	5.9	2.4	4.1
35.2	At3g59620	unknown protein	3.6	4.3	4.6
21.1	At2g33270	ACHT3 (ATYPICAL CYS HIS RICH THIOREDOXIN 3)	4.7	3.6	4.0
20.1.7	At3g48231	LCR48 (Low-molecular-weight cysteine-rich 48)	3.9	4.1	4.2
35.2	At3g50376	pseudogene of NLI interacting factor (NIF) family protein	4.1	4.2	3.9
35.2	At1g55221	pseudogene	4.6	3.6	3.3
31.4	At1g07725	ATEXO70H6 (exocyst subunit EXO70 family protein H6)	3.7	3.9	3.5
35.2	At2g17305	unknown protein	3.8	3.9	3.4
35.2	At4g29200	beta-galactosidase	3.5	3.8	3.9
33.99	At5g07930	MCT2 (mei2 C-Terminal RRM only like 2); nucleic acid binding protein	4.2	2.8	4.2
35.2	At5g45690	unknown protein	4.0	4.2	2.9
35.1	At4g33820	glycosyl hydrolase family 10 protein	4.2	4.4	2.5
27.3.24	At5g51860	MADS-box protein (AGL72)	3.9	3.9	3.2
27.3.7	At3g21880	zinc finger (B-box type) family protein	3.5	3.7	3.8
35.2	At1g24256	unknown protein	2.4	3.9	4.6
30.2.17	At4g11890	protein kinase family protein	1.7	4.5	4.5
35.2	At5g28295	unknown protein	3.6	3.4	3.5
35.1	At3g57840	self-incompatibility protein-related	3.1	3.7	3.6
20.1	At2g15040	ATRLP18, RECEPTOR LIKE PROTEIN 18, RLP18	4.4	2.2	3.7
29.5.11.4.3.2	At5g53840	F-box family protein (FBL13)	3.4	3.4	3.4
35.2	At2g11440	pseudogene	3.6	3.6	3.0
35.1	At3g48620	unknown protein; located in outer membrane	3.7	2.7	3.7
29.3.4.1	At2g38960	AERO2 (Arabidopsis endoplasmic reticulum oxidoreductins 2)	4.0	2.3	3.4
35.1	At4g26860	pyridoxal phosphate binding	3.7	2.9	3.1
29.5.11.4.3.2	At5g44220	F-box family protein	3.7	2.3	3.7
20.1.7	At4g09984	LCR34 (Low-molecular-weight cysteine-rich 34)	3.2	3.3	3.2
27.1	At5g03580	polyadenylate-binding protein, putative	3.4	3.4	2.9
35.2	At4g08022	pseudogene, hypothetical protein	3.4	3.4	2.8
35.2	At4g05018	unknown protein	3.7	3.1	2.7
33.99	At1g21890	nodulin MtN21 family protein	2.9	3.0	3.5
35.2	At1g23910	unknown protein; involved in response to biotic stimulus, defense response	3.0	3.6	2.9
24	At5g16080	AtCXE17 (Arabidopsis thaliana carboxyesterase 17)	3.5	2.9	3.0

35.2	At5g51090	unknown protein	3.3	2.9	3.2
33.99	At2g30300	nodulin-related protein	3.2	3.0	3.0
33.99	At2g37860	LCD1 (LOWER CELL DENSITY 1)	3.0	3.3	2.9
26.4.1	At3g24330	glycosyl hydrolase family 17 protein	2.9	3.0	3.1
35.1	At3g48209	thionin family protein	3.0	3.0	2.8
20.1.7.12	At4g14272	defensin-like (DEFL) family protein	3.1	2.9	2.7
35.1.19	At1g48590	C2 domain-containing protein	3.8	3.5	1.5
10.8.1	At1g11590	pectin methylesterase, putative	2.9	2.9	2.8
29.5.11.1	At5g48700	ubiquitin-related	2.5	3.4	2.6
35.2	At3g30520	unknown protein	2.8	2.8	2.8
11.1.8	At1g21540	AMP-dependent synthetase and ligase family protein	3.0	2.7	2.4
35.2	At3g43829	unknown protein	4.0	2.1	2.1
35.2	At5g29044	unknown protein	3.8	2.2	2.1
35.1	At3g58290	meprin and TRAF homology domain-containing protein	2.7	3.0	2.4
35.2	At5g35890	beta-galactosidase	3.8	1.5	2.5
35.2	At1g57906	unknown protein	2.7	2.4	2.8
26.3.2	At4g38590	glycosyl hydrolase family 35 protein	2.9	2.4	2.5
30.2.9	At1g24650	leucine-rich repeat family protein	2.3	2.6	2.9
27.3.24	At2g24840	AGL61 (AGAMOUS-LIKE 61); transcription factor	2.6	2.8	2.3
35.2	At3g42140	nucleic acid binding / zinc ion binding	2.4	1.8	3.5
35.1.41	At1g30795	hydroxyproline-rich glycoprotein family protein	2.9	2.4	2.3
35.2	At1g26350	unknown protein	3.4	1.2	3.0
29.5.7	At3g59990	MAP2B (METHIONINE AMINOPEPTIDASE 2B)	2.6	2.4	2.7
27.3.37	At1g67100	LBD40 (LOB DOMAIN-CONTAINING PROTEIN 40)	1.7	4.4	1.4
16.2	At1g32910	transferase family protein	2.8	2.0	2.6
26.10	At4g15310	CYP702A3; monooxygenase	1.4	3.5	2.6
27.3.67	At1g61320	unknown protein	2.4	2.2	2.8
35.2	At5g50360	unknown protein	2.1	2.5	2.8
35.2	At1g03240	unknown protein	2.5	2.5	2.3
35.1	At4g19910	Toll-Interleukin-Resistance (TIR) domain-containing protein	2.8	2.3	2.2
35.1	At3g06880	nucleotide binding protein	3.0	2.1	2.2
20.1.7	At5g42797	LCR28 (Low-molecular-weight cysteine-rich 28)	3.8	2.0	1.4
29.5.5	At3g52000	scpl36 (serine carboxypeptidase-like 36)	2.5	2.6	2.0
35.1	At5g52690	heavy-metal-associated domain-containing protein	2.4	2.4	2.2
26.10	At1g19630	CYP722A1; monooxygenase	2.5	2.5	1.9

35.2	At1g77655	unknown protein	3.0	1.9	2.1
33.99	At1g23240	caleosin-related family protein	1.1	2.9	2.9
35.2	At3g58300	unknown protein	2.3	2.3	2.3
28.1	At2g02650	reverse transcriptase-related protein	4.0	1.1	1.6
35.1	At1g68630	unknown protein	2.7	2.1	1.9
35.2	At3g43950	phosphotransferase, alcohol group as acceptor	2.2	2.3	2.1
29.2.1.2.2.30	At1g22110	structural constituent of ribosome	3.5	1.6	1.4
35.2	At5g05113	unknown protein	3.7	1.3	1.5
33.1	At1g03890	cupin family protein	2.1	2.5	1.8
27.3.6	At1g06170	basic helix-loop-helix (bHLH) family protein	1.8	2.3	2.3
35.2	At1g68600	unknown protein	2.3	1.9	2.1
20.2.3	At3g06760	response to water deprivation	2.2	1.8	2.4
34.2	At1g34580	carbohydrate transmembrane transporter	2.5	1.8	2.0
26.4.1	At3g57260	BGL2 (BETA-1,3-GLUCANASE 2); cellulase	1.4	3.3	1.6
35.2	At1g53285	unknown protein	2.3	2.3	1.8
35.1	At1g21928	thionin family protein	2.2	2.2	1.8
35.2	At3g27025	unknown protein	3.2	1.5	1.5
27.4	At1g67950	RNA recognition motif (RRM)-containing protein	2.4	2.3	1.5
35.1	At5g14980	esterase/lipase/thioesterase family protein	2.7	2.0	1.4
35.1	At1g31390	meprin and TRAF homology domain-containing protein	2.4	1.4	2.4
35.2	At4g10860	unknown protein	1.8	3.0	1.3
29.3.4.3	At3g13205	pseudogene of vacuolar protein sorting-associated protein	1.9	2.1	2.1
20.1.7.12	At1g33607	defensin-like (DEFL) family protein	1.5	2.2	2.5
35.2	At2g20625	unknown protein	2.5	2.2	1.4
29.3.4.99	At5g27220	protein transport protein-related	2.1	2.1	1.9
35.2	At1g04501	unknown protein	2.0	2.0	2.0
27.1	At1g61275	U12; snRNA	1.5	2.2	2.3
27.3.3	At5g25390	SHN2 (shine2); transcription factor	2.1	2.0	1.9
10.2.1	At4g23990	ATCSLG3; cellulose synthase	1.6	2.5	1.8
10.7	At5g39280	ATEXPA23 (EXPANSIN A23)	2.2	1.6	2.2
32	At2g19425	MIR156G; miRNA	2.4	1.9	1.6
26.3.4	At4g39000	AtGH9B17 (Arabidopsis thaliana glycosyl hydrolase 9B17)	2.3	1.5	2.1
30.3	At2g41860	CPK14; calmodulin-dependent protein kinase	2.0	2.0	2.0
35.2	At1g42710	unknown protein	1.1	2.5	2.3
34.7	At4g08878	inorganic phosphate transporter	1.9	2.0	1.9

27.3.24	At5g39810	AGL98 (AGAMOUS-LIKE 98); transcription factor	2.5	1.3	2.0
29.5.11.4.3.2	At3g22650	CEG (CEGENDUO)	1.1	2.3	2.4
35.1	At3g46190	meprin and TRAF homology domain-containing protein	1.3	3.1	1.4
35.2	At1g28375	unknown protein	1.9	1.9	1.9
26.2	At3g02100	UDP-glucoronosyl/UDP-glucosyl transferase family protein	2.3	2.2	1.1
31.1	At4g27360	dynein light chain, putative	2.3	1.3	2.0
35.2	At3g24640	lyase	3.1	1.2	1.2
35.2	At5g65166	unknown protein	2.8	1.4	1.2
35.2	At1g79770	unknown protein	1.8	1.9	1.7
35.2	At4g14260	unknown protein	2.1	1.6	1.8
31.2	At2g36250	FTSZ2-1; protein binding protein; structural molecule	3.0	1.0	1.4
27.1	At2g43810	small nuclear ribonucleoprotein F, putative / U6 snRNA-associated Sm-like protein, putative	1.9	2.1	1.3
27.3.55	At5g61070	HDA18; H3/H4 histone acetyltransferase/ histone deacetylase	1.9	1.7	1.8
29.2.7.1.2	At4g31075	pre-tRNA	1.2	2.1	2.1
35.1.5	At4g01400	unknown protein	1.7	1.9	1.6
17.8.1	At4g26420	GAMT1; S-adenosylmethionine-dependent methyltransferase/ gibberellin carboxyl-O-methyltransferase	2.0	1.4	1.9
26.18	At1g54980	invertase/pectin methylesterase inhibitor family protein	2.0	1.6	1.6
35.2	At1g32670	unknown protein	2.0	2.0	1.2
35.2	At1g54880	unknown protein	1.7	1.7	1.7
30.5	At5g54840	SGP1; GTP binding protein	1.7	1.8	1.7
35.1	At4g14730	LIFEGUARD 1, Bax inhibitor-1 family protein	2.4	1.0	1.7
32	At3g55734	MIR393B; miRNA	1.8	2.0	1.4
4.3.8	At3g43290	unknown protein	1.7	1.7	1.7
35.2	At4g06490	unknown protein	2.1	1.2	1.7
26.28	At5g15720	GLIP7; carboxylesterase/ lipase	2.3	1.4	1.4
26.21	At1g64235	protease inhibitor/seed storage/lipid transfer protein (LTP) family protein	2.2	1.3	1.6
26.10	At4g15440	HPL1 (HYDROPEROXIDE LYASE 1)	2.1	1.5	1.4
34.16	At2g29940	PDR3 (PLEIOTROPIC DRUG RESISTANCE 3); ATPase, coupled to transmembrane movement of substances	2.2	1.0	1.7
35.2	At2g14405	pseudogene	1.8	1.6	1.6
34.99	At3g03620	MATE efflux family protein	2.1	1.5	1.4
35.1.25	At1g24220	paired amphipathic helix repeat-containing protein	1.9	1.1	2.0

29.4	At1g01450	protein kinase-related	1.6	1.2	2.1
16.2	At2g19070	SHT (SPERMIDINE HYDROXYCINNAMOYL TRANSFERASE)	1.9	1.1	1.9
29.5.11.4.3.2	At3g46050	kelch repeat-containing F-box family protein	1.8	1.6	1.4
35.2	At3g26235	unknown protein	2.0	1.3	1.5
26.3	At2g04060	beta-galactosidase, putative	1.8	1.2	1.8
35.2	At5g24060	unknown protein	1.2	1.8	1.8
30.2.17	At4g28670	protein kinase family protein	1.7	1.5	1.6
10.8.1	At3g27980	pectinesterase family protein	1.8	1.8	1.2
29.5.11.4.3.2	At4g11770	kelch repeat-containing F-box family protein	1.9	1.7	1.2
10.2.1	At2g25540	CESA10; cellulose synthase 10	1.8	1.4	1.5
29.2.7.1.2	At4g34035	pre-tRNA	2.1	1.1	1.5
26.10	At3g26270	CYP71B25; monooxygenase	1.8	1.3	1.6
35.1	At1g47880	pseudogene, similar to NL0E, similar to disease resistance protein	1.6	1.6	1.5
35.2	At1g68380	unknown protein	1.9	1.5	1.3
35.2	At2g10920	unknown protein	1.5	1.5	1.6
31.1	At4g03450	ankyrin repeat family protein	1.7	1.9	1.0
17.6.1.11	At5g07200	YAP169; gibberellin 20-oxidase	2.3	1.2	1.1
16.5.1.3.2.1	At1g54040	ESP (EPITHIOSPECIFIER PROTEIN); enzyme regulator	1.6	1.3	1.7
27.3.41	At4g31610	REM1 (REPRODUCTIVE MERISTEM 1); transcription factor	1.8	1.2	1.5
27.3.37	At1g65620	AS2 (ASYMMETRIC LEAVES 2)	1.5	2.0	1.0
20.1.7.12	At2g42885	defensin-like (DEFL) family protein	1.6	1.3	1.6
29.5.11.4.3.2	At3g25460	F-box family protein	1.6	1.1	1.8
28.1	At2g42320	nucleolar protein gar2-related protein	2.2	1.1	1.2
20.1.7.12	At5g44430	PDF1.2c (plant defensin 1.2c)	1.7	1.7	1.1
17.7.1.10	At1g19640	JMT (JASMONIC ACID CARBOXYL METHYLTRANSFERASE); jasmonate O-methyltransferase	1.9	1.1	1.4
29.5.3	At5g28235	Ulp1 protease family protein	1.6	1.2	1.6
29.2.2.50	At2g06822	unknown protein	1.9	1.4	1.1
27.3.3	At1g12890	AP2 domain-containing transcription factor, putative	1.5	1.6	1.4
27.3.30	At1g11850	unknown protein	1.4	1.5	1.5
35.1	At3g52620	unknown protein	1.4	1.5	1.5
27.3.25	At2g32460	MYB101	1.4	2.0	1.1
35.2	At5g54067	unknown protein	1.4	1.5	1.4
27.3.24	At3g61120	AGL13 (AGAMOUS-LIKE 13); transcription factor	1.8	1.2	1.3
29.5.11.4.3.2	At3g17560	F-box family protein	1.3	1.3	1.7

35.2	At1g34392	unknown protein	1.4	1.5	1.3
27.3.32	At1g55600	WRKY10	1.3	1.5	1.4
35.1	At2g31005	cysteine-rich peptide family protein	1.5	1.6	1.2
29.5.11.4.3.2	At1g55070	F-box family protein	1.7	1.2	1.3
35.2	At3g45460	zinc ion binding protein	1.7	1.3	1.3
35.2	At1g23074	unknown protein	1.7	1.2	1.3
26.13	At1g56360	PAP6 (PURPLE ACID PHOSPHATASE 6)	1.9	1.1	1.3
29.5.11.4.3.2	At1g16940	F-box family protein	1.4	1.7	1.1
35.1.19	At1g20080	SYNAPTOTAGMIN 2	2.0	1.0	1.1
35.1.42	At3g51290	proline-rich family protein	1.7	1.0	1.4
31.3	At4g30820	cyclin-dependent kinase-activating kinase assembly factor-related / CDK-activating kinase assembly factor-related	1.5	1.5	1.1
31.2	At1g19810	putative cell division control protein	1.6	1.0	1.4
27.3.99	At5g45113	mitochondrial transcription termination factor-related protein	1.4	1.4	1.2
20.2	At2g45130	SPX3 (SPX DOMAIN GENE 3)	1.8	1.0	1.1
27.3.67	At1g75560	zinc knuckle (CCHC-type) family protein	1.0	1.7	1.2
27.3.83	At3g52910	AtGRF4 (GROWTH-REGULATING FACTOR 4)	1.3	1.4	1.2
8.1.7	At5g09600	SDH3-1; succinate dehydrogenase	1.2	1.3	1.3
35.2	At5g64541	unknown protein	1.4	1.3	1.1
35.1	At5g63225	glycosyl hydrolase family protein 17	1.3	1.1	1.3
35.1	At1g24147	unknown protein	1.3	1.1	1.4
26.11.1	At1g09510	cinnamyl-alcohol dehydrogenase family / CAD family	1.3	1.3	1.0
26.28	At2g04570	GDSL-motif lipase/hydrolase family protein	1.3	1.4	1.1
29.5.11.4.2	At1g68940	armadillo/beta-catenin repeat protein / U-box domain-containing protein	1.2	1.2	1.2
35.2	At2g19000	unknown protein; located in endomembrane system	1.1	1.1	1.5
35.2	At2g16668	unknown protein	1.4	1.2	1.0
29.2.3	At5g01940	eukaryotic translation initiation factor 2B family protein	1.5	1.0	1.1
35.2	At3g20720	unknown protein	1.4	1.1	1.1
35.1	At4g12890	gamma interferon responsive lysosomal thiol reductase family protein	1.3	1.2	1.0
35.2	At4g33380	unknown protein	1.1	1.2	1.1
35.2	At5g26730	unknown protein, located in mitochondrion	1.2	1.0	1.2
29.5.11.4.3.2	At5g40680	kelch repeat-containing F-box family protein	1.1	1.1	1.2
35.2	At2g41600	unknown protein; located in mitochondrial matrix	1.2	1.1	1.0
27.4	At5g24440	CID13 (CTC-Interacting Domain 13)	1.0	1.1	1.1

35.2	At1g22651	unknown protein	1.0	1.0	1.1
35.1.12	At5g09610	APUM21 (Arabidopsis Pumilio 21); RNA binding protein	1.0	1.0	1.0
32	At1g26975	MIR395B; miRNA	1.0	1.0	1.0
20.1	At1g31580	ECS1, CXC750	-1.1	-1.0	-1.3
29.3.4.3	At4g06498	pseudogene, vacuolar sorting receptor -related	-1.1	-1.2	-1.1
30.4	At3g59770	SAC9; inositol or phosphatidylinositol phosphatase	-1.2	-1.1	-1.1
31.1	At4g25590	ADF7 (actin depolymerizing factor 7)	-1.3	-1.2	-1.1
35.2	At1g26720	unknown protein	-1.3	-1.2	-1.2
35.2	At2g15327	unknown protein	-1.2	-1.4	-1.0
35.2	At2g13550	unknown protein	-1.7	-1.1	-1.0
1.1.1.1	At2g40100	LHCB4.3 (light harvesting complex of photosystem II)	-1.2	-1.1	-1.5
27.3.16	At5g50490	NF-YC5 (NUCLEAR FACTOR Y, SUBUNIT C5)	-1.2	-1.3	-1.4
35.2	At4g11930	unknown protein	-1.6	-1.2	-1.3
35.2	At1g47980	unknown protein	-1.2	-1.5	-1.5
27.3.57	At1g30810	transcription factor	-1.7	-1.2	-1.5
35.2	At1g54600	pseudogene, hypothetical protein	-1.0	-2.3	-1.1
33.99	At1g24520	BCP1 | BCP1	-1.8	-1.8	-1.1
10.8.1	At2g47040	VGD1 (VANGUARD1)	-1.3	-2.4	-1.0
35.1	At5g08000	E13L3, PDCB2	-1.5	-2.2	-1.1
27.1	At3g14735	U6-1; snRNA	-1.8	-1.7	-1.3
27.3.24	At1g28460	Symbols: AGL59 | AGL59 (AGAMOUS-LIKE 59)	-1.1	-1.7	-2.0
35.2	At1g35614	unknown protein	-1.8	-1.3	-1.7
30.5	At3g16130	ROPGEF13; Rho guanyl-nucleotide exchange factor	-1.5	-1.9	-1.5
4.1.6	At5g28917	pseudogene	-1.3	-2.3	-1.5
26.6	At3g53140	O-diphenol-O-methyl transferase, putative	-1.0	-2.8	-1.3
26.18	At5g50040	invertase/pectin methylesterase inhibitor family protein	-1.3	-2.8	-1.0
35.1	At2g32870	meprin and TRAF homology domain-containing protein	-2.2	-2.0	-1.1
35.2	At2g15840	pseudogene, hypothetical protein	-1.4	-1.8	-2.1
35.2	At1g43415	unknown protein	-1.5	-2.8	-1.1
35.2	At1g55370	NDF5 | carbohydrate binding / catalytic	-1.2	-2.8	-1.5
9.1.1	At5g44140	ATPHB7 (PROHIBITIN 7)	-1.0	-3.1	-1.4
20.1.7.12	At3g05730	defensin-like (DEFL) family protein	-1.1	-3.8	-1.0
26.10	At5g35715	CYP71B8 | CYP71B8	-1.7	-3.8	-1.3
35.1	At2g03972	pseudogene of heat shock protein	-3.4	-2.0	-1.5
35.2	At3g10116	unknown protein	-1.6	-2.1	-3.3

20.1.7	At5g43740	disease resistance protein (CC-NBS-LRR class), putative	-3.7	-1.3	-2.0
17.6.3	At1g22690	gibberellin-responsive protein, putative	-4.1	-1.8	-1.1
35.2	At4g20520	RNA binding / RNA-directed DNA polymerase	-2.5	-2.3	-2.5
30.2.99	At4g20650	molecular_function unknown	-2.5	-1.6	-3.3
35.2	At3g61826	unknown protein	-1.4	-4.1	-2.1
27.3.6	At2g34820	basic helix-loop-helix (bHLH) family protein	-2.9	-3.6	-1.2
35.2	At5g36770	unknown protein	-1.4	-4.5	-2.1
29.5.11.4.2	At5g41450	zinc finger (C3HC4-type RING finger) family protein	-4.8	-2.2	-1.6
35.2	At5g06755	unknown protein	-3.5	-3.4	-1.7
20.1	At4g09940	avirulence-responsive family protein	-3.2	-4.2	-1.2
35.1	At1g10717	Maternally expressed gene (MEG) family protein	-3.9	-3.8	-1.1
35.2	At4g08874	unknown protein	-6.0	-2.9	-1.9
35.2	At1g53480	Symbols: ATMRD1, MRD1	-1.9	-3.2	-6.1
17.1.1	At2g36020	HVA22J (HVA22-LIKE PROTEIN J)	-4.2	-7.1	-2.2

Table S3. Primers used in this study

GAPDH:
LP: GAGCTGACTACGTTGTTGAG
RP: GGAGACAATGTCAAGGTCGG

WRKY6 **(Salk line):**
LP**:** GAACGTATTAGCCAATCACGC
RP**:** TGTGGACGTGTCATAATTTGG

whWRKY6
LP**:** GTTGTTTCCTTCGCCGTCGTGG
RP**:** ATGGACAGAGGATGGTCTGGTC

PHO1
LP**:** AGACAACCGGGTGTTACTTCTTGG
RP**:** GTCCCACCATCTCCTTACACATTG

EF-H
LP**:** CGCAGAATACAAGGAGGCC
RP**:** CGTATCGTAGCTCGCCTGC

AT2G32830
LP: AGCCAAAACGCAGATGTACC
RP: AAGGCCACACCATTAACAGC

AT4G08878
LP: CCGAAAACCGTCATGGTAAC
RP: TTGCATAGCAAAAACCGATG

AT1G08090
LP: GAACTTTGGATCAGGGCTCA
RP: AAGAACATGCTTCCCCATTG

AT1G08100
LP: GACATTGGAAACGCTGGAGT
RP: CACGAAGCTCATGGAGAACA

AT5g44430
LP: CTTGTGTGCTGGGAAGACATA
RP: AGCACAGAAGTTGTGCGAGAA

At5g30500
LP: TCCCTTCGACGTACAACCTC
RP: TCTCGGTCCATATGCTCCTC

AT5G62850
LP: CGACAGTCTCCTCGTCATCA
RP: CCACCGCCATGAATATTACC

AT1G01980
LP: CCAACCCTACGTGCCTACAT
RP: GCGAGTCTTGAGCTGGAAGT

AT2G19425
LP: AACGAAGGCGACAGAAGAGA
RP: GAGAGCAGGAGACAAGAAGAGAG

AT5G61070
LP: GATGGAGAACTGGAGGCAAG
RP: GCTCCTGCTGCAAACTCTCT

AT1G02550
LP: TCGTCTCAATCCTCCCACTC
RP: GGCGTTGACTTATCCACGAT

AT5G52300
LP: TGCAGAGGAAGGAAAAGGTG
RP: ATCCGAAAACCCCATAGTCC

Table S4. Microarray validation with Real-time PCR

	RT PCR	Microarray
**At5g62850**	4.3 ± 0.01	4.7
**At1g01980**	3.6 ± 0.7	4.2
**At2g19425**	2.2 ± 0.3	2.4
**At5g61070**	1.8 ± 0.6	1.9
**At1g08090**	1.4 ± 0.1	1.1
**At1g08100**	1.5 ± 0.02	1.1
**At5g30500**	2.5 ± 0.2	2.9
**At4g08878**	1.8 ± 0.4	1.9
**At5g44430**	1.2 ± 0.5	1.7
**At2g32830**	1.4 ± 0.08	1.6
